# Friedewald and Martin–Hopkins formulae for estimating low-density lipoprotein cholesterol in a Malagasy population

**DOI:** 10.4102/ajlm.v15i1.3012

**Published:** 2026-02-09

**Authors:** Faralahy H. Rakotonjafiniarivo, Tokinomenjanahary Antsonantenaina, Mahefa S. Rakotomalala, Rajo D. Andriambelo, Miora K. Ranaivosoa

**Affiliations:** 1 Department of Biology, Faculty of Medicine, Antananarivo University, Antananarivo, Madagascar; 2 Faculty of Medicine, Antananarivo University, Antananarivo, Madagascar

**Keywords:** low-density lipoprotein cholesterol, triglyceride, Friedewald formula, Martin–Hopkins formula, Madagascar

## Abstract

**Background:**

Low-density lipoprotein cholesterol (LDL-C) estimation is commonly used in Madagascar due to cost-effectiveness. However, genetic variability and formula limitations may affect accuracy.

**Objective:**

To compare LDL-C estimated by the Friedewald and Martin–Hopkins formulae with directly measured LDL-C in a Malagasy population.

**Methods:**

LDL-C values estimated using both formulae were compared with direct LDL-C in 346 samples from patients ≥ 18 years analysed in a biochemistry laboratory. Samples were divided into four groups based on triglyceride levels: < 1.13 mmol/L; 1.13 mmol/L – 1.69 mmol/L; 1.69 mmol/L – 2.26 mmol/L; ≥ 2.26 mmol/L.

**Results:**

Both formulae showed a strong, statistically significant correlation with direct LDL-C (*r* = 0.89). Mean comparison revealed overestimation by both formulae, more pronounced with Friedewald (mean difference 0.15 mmol/L) than Martin–Hopkins (0.21 mmol/L). Differences increased with rising triglyceride levels. Both formulae demonstrated good agreement with direct measurement, acceptable biases and similar limits, but Friedewald had a lower overall percentage error.

**Conclusion:**

The Friedewald formula showed better correlation, higher concordance and lower mean difference than Martin–Hopkins. Both formulae showed limitations depending on triglyceride concentration.

**What this study adds:**

This study evaluates Friedewald and Martin–Hopkins LDL-C estimation against direct measurement in a Malagasy population, highlighting their validity in Africa and implications for clinical decisions in resource-limited settings.

## Introduction

According to the World Health Organization, cardiovascular disease is the leading cause of death worldwide.^1^ Among the main risk factors for these diseases, hypercholesterolaemia and, more specifically, elevated low-density lipoprotein cholesterol (LDL-C), plays a major role in the onset of atherosclerosis and cardiovascular events.^2,3^ Determining low-density lipoprotein (LDL) levels is important in initiating treatment and monitoring dyslipidaemia.^4^ Reducing LDL-C through lipid-lowering treatments decreases the risk of myocardial infarction and stroke significantly.^5^ An accurate LDL-C level is crucial for the diagnosis and management of patients with dyslipidaemia.^6^ Ultracentrifugation is the gold standard method for analysing lipids and lipoproteins, but it is time consuming, expensive, and requires sophisticated equipment.^7^ Other methods allow direct measurement of LDL, but in routine laboratories in a resource-limited country such as Madagascar, they are rarely available. Owing to these limitations, many laboratories use formulae to estimate LDL-C, with the Friedewald formula being the most commonly used in most Malagasy laboratories. This study evaluates the accuracy of the Friedewald and Martin–Hopkins formulae for estimating LDL-C by comparing their results with directly measured LDL-C at different serum triglyceride levels.

## Methods

### Ethical consideration

This study followed all ethical standards for research without direct contact with human or animal subjects. The study was conducted using retrospective data obtained from routine laboratory records at the biochemistry laboratory of a tertiary care hospital in Madagascar. All data were anonymised fully prior to analysis, and no patient identifiers were included. According to institutional policy and national regulations, retrospective studies based on anonymised laboratory data do not require formal approval from an ethics committee. The study was conducted in accordance with the principles of the Declaration of Helsinki.

### Study design

This is a retrospective observational study comparing the performance of LDL cholesterol estimation methods (Friedewald and Martin–Hopkins formulae) with the direct measurement method. The study population consists of samples from patients who underwent a complete lipid profile (total cholesterol, high-density lipoprotein cholesterol [HDL-C], LDL-C, and triglycerides) at the biochemistry laboratory of a tertiary care hospital in Madagascar, over a period of two years (from January 2023 to December 2024). Biological tests from subjects under the age of 18 and having triglyceride values above 4.52 mmol/L (400 mg/dL) were excluded. The samples were classified into four groups according to triglyceride levels: (1) Group 1, < 1.13 mmol/L; (2) Group 2, 1.13 mmol/L – 1.69 mmol/L; (3) Group 3, 1.69 mmol/L – 2.26 mmol/L; and (4) Group 4 ≥ 2.26 mmol/L. Group 1 represents very low triglyceride values, while Group 2 corresponds to optimal, Group 3 to borderline-high, and Group 4 to high to very high triglyceride values, according to the American Heart Association and National Cholesterol Education Program.

### Lipoprotein assay

Plasma concentrations of cholesterol, HDL-C, triglycerides, and LDL-C were measured on a Mindray BS300® biochemistry analyser (Mindray Bio-Medical Electronics Co. Ltd, Shenzhen, China) with Cromatest® reagent kits (Linea Chemicals S.L., Barcelona, Spain).

The measurements of total cholesterol and triglycerides are based on colorimetric enzymatic methods. The HDL-C assay using Cromatest® reagents is performed via a selective precipitation method.

The direct method for quantifying LDL-C is based on modified polyvinyl sulfonic acid and polyethylene glycol methyl ether associated with the classic precipitation method in optimised ratios of polyvinyl sulfonic acid and polyethylene glycol methyl ether and selected detergents.

The analytical performance of the direct LDL-C assay was: intra-assay coefficient of variation (CV) 0.67%, inter-assay CV 2.87%, linearity up to 25.60 mmol/L, limit of detection 0.17 mmol/L, and bias < 4% versus reference method. The assay showed no significant interference from haemolysis (500 mg/dL), bilirubin (20 mg/dL), and ascorbic acid (50 mg/dL) within the manufacturer’s limits.

### Low-density lipoprotein cholesterol estimation formulae

#### Friedewald formula

For units in mmol/L (see Equation 1):


$$\begin{array}{l} \text{LDL-C-F}=Total\;Cholesterol-High\;Density\;Lipoprotein\\ Cholesterol-\left(\frac{Triglyceride}{2.2}\right) \end{array}$$ [Eqn 1]

For units in mg/dL (see Equation 2):


$$\begin{array}{l} \text{LDL-C-F}=Total\;Cholesterol-High\;Density\;Lipoprotein\\ Cholesterol-\left(\frac{Triglyceride}{5}\right) \end{array}$$ [Eqn 2]

#### Martin–Hopkins formula

Units must be in mg/dL (see Equation 3):


$$\begin{array}{l} \text{LDL-C-M}=Total\;Cholesterol-High\;Density\;Lipoprotein\\ Cholesterol-\left(\frac{Triglyceride}{Adjustment\;coefficient}\right) \end{array}$$ [Eqn 3]

The adjustment coefficient is determined based on the HDL-C and triglyceride values, with each observation being associated with one of 180 predefined correction factors.

For this study, the LDL-C value according to the Martin–Hopkins formula was calculated according to the guidelines found on the website (http://www.ldlcalculator.com) and then converted back to mmol/L.

### Unit conversion

For total cholesterol, HDL-C, and LDL-C: 1 mmol/L = 38.67 mg/dL

For triglyceride: 1 mmol/L = 88.57 mg/dL

### Statistical analyses

The programming and statistical analysis language R version 4.5.1 (The R Foundation for Statistical Computing, Vienna, Austria) was used for data analysis. The mean, median, standard deviation and interquartile range were used to describe the data. Pearson’s correlation test was used to evaluate the relationship between directly measured and calculated values, while Student’s paired *t*-test was used to determine the presence of significant differences between the methods. The Bland-Altman plot was used to determine the agreement between the LDL-C estimation techniques and direct measurement. A *p*-value < 0.05 was used as the significance threshold.

## Results

This study included 346 blood samples. The median age of the subjects was 56 years (interquartile range: 46, 64; mean ± standard deviation: 54.22 ±14.67 years), of whom 181 (52.3%) were women, giving a gender ratio of 0.86 (Table 1). Table 2 shows the mean values of the lipid profile components obtained by direct measurement and the mean values of LDL-C estimated using the Friedewald and Martin–Hopkins formulae.

**TABLE 1:** Characteristics of the study population in study samples in Antananarivo, Madagascar, from January 2023 to December 2024.

Variable	Category	Overall	Group 1	Group 2	Group 3	Group 4
Age (years)	Median	56	57	55	56	56
	Interquartile range (Q1, Q3)	45, 64	43, 65	45, 65	49, 61	47, 62
Gender	Female	181	82	47	24	28
	Male	165	57	44	31	33

Q1, first quartile (25^th^ percentile); Q3, third quartile (75^th^ percentile).

**TABLE 2:** Lipid profiles (total cholesterol, low-density lipoprotein cholesterol, high-density lipoprotein cholesterol, triglycerides) in study samples in Antananarivo, Madagascar, from January 2023 to December 2024.

Variable	Mean	s.d.
Total cholesterol	4.52	1.47
HDL-C	1.01	0.35
Triglycerides	1.49	0.82
LDL-C-D	2.68	1.08
LDL-C-F	2.83	1.25
LDL-C-M	2.89	1.23

HDL-C, high-density lipoprotein cholesterol; LDL-C-D, low-density lipoprotein cholesterol direct measurement; LDL-C-F, low-density lipoprotein cholesterol Friedewald calculation; LDL-C-M, low-density lipoprotein cholesterol Martin–Hopkins calculation; s.d., standard deviation.

**FIGURE 1:**
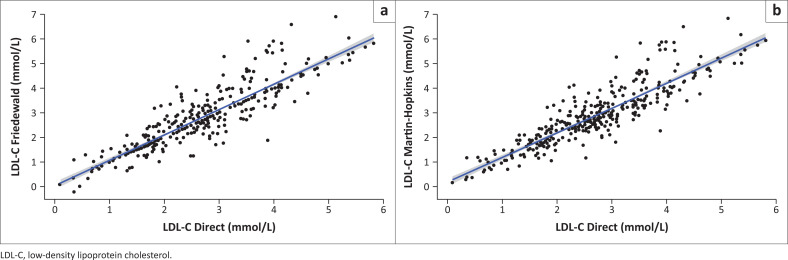
Correlation between direct low-density lipoprotein cholesterol and calculated low-density lipoprotein cholesterol using (a) Friedewald (*r* = 0.89, *p* < 0.001) and (b) Martin–Hopkins (*r* = 0.89, *p* < 0.001) formulae.

Paired correlation tests determined an identical Pearson coefficient of 0.89 for the Friedewald and Martin–Hopkins formulae (Figure 1). Similar results were observed for the groups according to triglyceride levels (Table 3).

**TABLE 3:** Correlation between direct low-density lipoprotein cholesterol and calculated high-density lipoprotein cholesterol using different formulae, assessed by Karl Pearson’s correlation method in study samples in Antananarivo, Madagascar, from January 2023 to December 2024.

Sample groups	Methods	*r*	*p*	Lower 95% CI	Upper 95% CI
Group 1	Friedewald formula	0.88	< 0.001	0.83	0.91
	Martin–Hopkins formula	0.88	< 0.001	0.83	0.91
Group 2	Friedewald formula	0.88	< 0.001	0.83	0.92
	Martin–Hopkins formula	0.88	< 0.001	0.83	0.92
Group 3	Friedewald formula	0.89	< 0.001	0.81	0.93
	Martin–Hopkins formula	0.88	< 0.001	0.81	0.93
Group 4	Friedewald formula	0.90	< 0.001	0.83	0.94
	Martin–Hopkins formula	0.89	< 0.001	0.83	0.94

CI, confidence interval.

Comparison of the means using a paired *t*-test (Table 4) revealed that the LDL-C levels calculated using both formulae are overestimated compared to the levels measured by direct assay, with a mean difference of 0.15 mmol/L for the Friedewald formula, and 0.21 mmol/L for the Martin–Hopkins formula. The same is true for the subgroups, with an increasing average difference as triglyceride levels rise. The non-significant differences concern the Martin–Hopkins formula for triglyceride level < 1.13 mmol/L (Group 1) and the Friedewald formula for triglyceride level > 2.26 mmol/L (Group 4).

**TABLE 4:** Paired *t*-test of low-density lipoprotein cholesterol values in study samples in Antananarivo, Madagascar, from January 2023 to December 2024.

Comparison	Mean difference† (mmol/L)	Lower95% CI	Upper95% CI	*t*	*p*
**Total**					
LDL-C-D and LDL-C-F	0.15	0.09	0.21	4.74	< 0.001*
LDL-C-D and LDL-C-M	0.21	0.15	0.27	6.88	< 0.001*
**Group 1**					
LDL-C-D and LDL-C-F	0.12	0.03	0.03	2.74	0.007*
LDL-C-D and LDL-C-M	0.07	−0.02	−0.02	1.55	0.120
**Group 2**					
LDL-C-D and LDL-C-F	0.19	0.07	0.07	3.25	0.002*
LDL-C-D and LDL-C-M	0.22	0.11	0.11	3.95	< 0.001*
**Group 3**					
LDL-C-D and LDL-C-F	0.23	0.06	0.06	2.71	0.009*
LDL-C-D and LDL-C-M	0.35	0.19	0.19	4.37	< 0.001*
**Group 4**					
LDL-C-D and LDL-C-F	0.08	−0.10	−0.10	0.86	0.390
LDL-C-D and LDL-C-M	0.39	0.22	0.22	4,69	< 0.001*

LDL-C-D, low-density lipoprotein cholesterol direct measurement; LDL-C-F, low-density lipoprotein cholesterol Friedewald calculation; LDL-C-M, low-density lipoprotein cholesterol Martin–Hopkins calculation.

* , statistically significant association.

† , Mean difference = Calculated LDL-C – Directly measured LDL-C.

**FIGURE 2:**
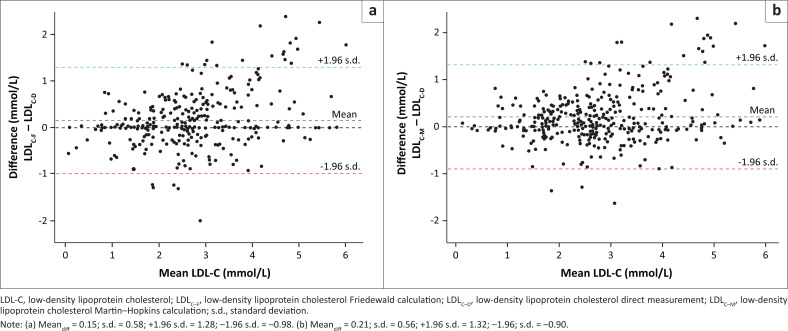
Bland-Altman plot between direct low-density lipoprotein cholesterol and calculated low-density lipoprotein cholesterol using (a) Friedewald and (b) Martin–Hopkins formulae.

Low-density lipoprotein cholesterol estimates using the Friedewald and Martin–Hopkins formulae show good agreement with the direct LDL-C measurement method, with acceptable biases and similar limits. The mean biases were 0.15 mmol/L for the Friedewald formula, and 0.21 mmol/L for the Martin–Hopkins formula (Figure 2). The percentage errors are reported in Table 5.

**TABLE 5:** Percentage of error for low-density lipoprotein cholesterol estimation techniques in study samples in Antananarivo, Madagascar, from January 2023 to December 2024.

Group	Friedewald formula	Martin–Hopkins formula
Overall	5.89	9.75
Group 1	6.81	4.23
Group 2	9.30	12.27
Group 3	7.65	13.55
Group 4	−2.89	15.17

## Discussion

Dyslipidaemia is a major cardiovascular risk factor, so accurate estimation of LDL-C is essential to ensure appropriate patient management. However, β quantification, which is the gold standard method for measuring LDL-C, remains difficult to access in routine practice as a result of its cost and technical complexity. As a result, laboratories generally use more affordable methods, such as direct measurement or estimation using mathematical formulae. Several formulae have been proposed for estimating LDL-C, each with varying degrees of accuracy depending on the characteristics of the population studied.^8,9,10,11^

This study focuses specifically on two of the most commonly used formulae:^12,13^ those of Friedewald and Martin–Hopkins, in order to evaluate their performance on a Malagasy population.

The median age of the subjects included in this study was 56 years (interquartile range: 46, 64), which is comparable to the results reported by Samuel et al., who analysed the lipid profiles of more than 5 million individuals (mean age ± standard deviation: 56 ± 16 years).^3^ However, this age is significantly higher than that observed in studies conducted by Farheen et al. (40.9 ± 8 years) and Krishnaveni et al. (48.4 ± 14.9 years) in India.^14,15^ It is well established that blood lipid levels tend to increase with age.^16^ The study population included a slightly higher proportion of women (52.3%), which is similar to the trend observed by Lam et al., where women accounted for 54% of the sample.^17^ Significant differences were observed between men and women regarding LDL-C levels. On average, LDL-C levels were higher in women.^18^

In the present study, a significant linear association between LDL-C calculation methods and the direct measurement method, with an identical Pearson correlation coefficient of 0.89, was found for both formulae. Although this trend is consistent with many previous studies, the strength of this correlation is slightly lower than that generally reported.^14,15^ Unlike most studies, where LDL-C is often underestimated using estimation formulae,^10,13,14,19^ this study showed a general tendency to overestimate LDL-C levels using both methods. Results similar to those of this study have been reported in India in individuals with hypothyroidism,^20^ and specifically for the Friedewald formula in an Iranian population.^21^

In this study, the Friedewald method has a slight advantage with a lower bias (0.15 mmol/L) compared to the Martin–Hopkins formula (0.21 mmol/L). These values are higher than those reported in Portugal, where the biases were 0.04 mmol/L for Friedewald and 0.03 mmol/L for Martin–Hopkins.^19^ These differences highlight the importance of considering not only the formulae used, but also the characteristics of the population concerned.

An increasing average difference was found in this study as triglyceride levels rose. The non-significant differences concern the Martin–Hopkins formula for triglyceride levels < 1.13 mmol/L (Group 1) and the Friedewald formula for triglyceride levels > 2.26 mmol/L (Group 4). In practice, it is important to remain vigilant about the limitations of using LDL-C estimation formulae. In our context, and in cases where direct measurement is not available, the Friedewald formula could be the most appropriate alternative, as it has a slight advantage with a lower bias (0.15 mmol/L) and a low percentage of error in the study population as a whole. The study by Farheen et al. found that the Martin–Hopkins formula was the best alternative to direct LDL-C measurement in an Indian population.^14^ Another study conducted in South Africa concluded that the Martin–Hopkins formula offered an advantage over the Friedewald formula in their study population and compared with the biochemistry analyser that was used.^22^ Given this divergence, some authors have developed recent LDL-C estimation formulae specific to a given population and triglyceride level.^23,24^

### The main strengths of the study

The study evaluates LDL-C estimation formulae (Friedewald and Martin–Hopkins) in a Malagasy population, providing local and relevant data for Africa. The findings have direct clinical applicability, helping resource-limited laboratories choose the most appropriate LDL-C method.

### Limitations

This study has some limitations. The sample size may limit the generalisability of the findings, and LDL estimation formulae can be less accurate at high triglyceride levels or in atypical lipid profiles. Analytical variability of direct LDL measurements and pre-analytical factors may have influenced the results. The clinical implication of discrepancies between calculated and directly measured LDL were not assessed.

### Conclusion

This study showed a good correlation between the Friedewald and Martin–Hopkins formulae and direct LDL-C measurement in a Malagasy population. However, a tendency towards overestimation was observed with both formulae, but more so with the Friedewald formula, as an alternative to direct measurement. Nevertheless, these results encourage further studies, including other population subgroups in order to evaluate the most appropriate formulae.
